# Disparities in Bystander Cardiopulmonary Resuscitation Performed by a Family Member and a Non-family Member

**DOI:** 10.2188/jea.JE20200068

**Published:** 2021-04-05

**Authors:** Nobuhiro Sato, Tasuku Matsuyama, Tetsuhisa Kitamura, Yasuo Hirose

**Affiliations:** 1 Department of Emergency and Critical Care Medicine, Niigata City General Hospital, Niigata, Japan; 2 Department of Emergency Medicine, Kyoto Prefectural University of Medicine, Kyoto, Japan; 3 Division of Environmental Medicine and Population Services, Department of Social and Environmental Medicine, Graduate School of Medicine, Osaka University, Osaka, Japan

**Keywords:** bystander cardiopulmonary resuscitation, cardiopulmonary resuscitation, out-of-hospital cardiac arrest

## Abstract

**Background:**

Although bystander cardiopulmonary resuscitation (BCPR) plays an essential role in out-of-hospital cardiac arrest (OHCA) care, little is known about the bystander-patient relationship in the actual setting. This study aimed to assess the disparities in BCPR performed by a family member and that performed by a non-family member.

**Methods:**

This population-based observational study involved all adult patients with witnessed OHCAs of medical origin in Niigata City, Japan, between January 2012 and December 2016, according to the Utstein style. We used logistic regression analysis to assess the association between the witnessing person and the probability of providing BCPR. Next, among those who received BCPR, we sought to investigate the difference between BCPR performed by family and that performed by non-family members in terms of whether those who witnessed the arrests actually performed BCPR.

**Results:**

During the study period, 818 were eligible for this analysis, with 609 (74.4%) patients witnessed by family and 209 (25.6%) patients witnessed by non-family members. Multivariable logistic regression analysis showed that OHCA patients witnessed by family were less likely to receive BCPR compared to those witnessed by non-family members (260/609 [42.7%] versus 119/209 [56.9%], *P* = 0.017). Among the witnessed patients for whom BCPR was performed, the proportion of BCPR actually performed by a family member was lower than that performed by a non-family member (242/260 [93.1%] versus 116/119 [97.5%], *P* = 0.011).

**Conclusions:**

In this community-based observational study, we found that a witnessing family member is less likely to perform BCPR than a witnessing non-family member.

## INTRODUCTION

Out-of-hospital cardiac arrest (OHCA) is a major public health problem around the world.^1^^,^^2^ After improvements in “chain of survival” linkages, the survival rate after an OHCA has continued to increase,^1^^,^^3^^,^^4^ but the probability of survivors after OHCA still remains low.^5^^–^^7^

Bystander cardiopulmonary resuscitation (BCPR) is a part of the “chain of survival” and plays an essential role in OHCA cares.^8^^,^^9^ However, according to previous studies, about half of OHCA patients did not receive BCPR.^4^^,^^7^ Therefore, to further improve the number of patients receiving BCPR, it is important to identify the obstacles and overcome them to increase BCPR. A previous study showed that women were less likely to receive BCPR than were men, regardless of age, when witnessed by a non-family member at a public location.^10^ Another nationwide study revealed that patients with OHCAs witnessed by family members were less likely to receive BCPR.^11^ Importantly, these studies did not include the data of who actually administered BCPR.

We, therefore, hypothesized that witnessing family member were less likely to actually perform BCPR than non-family members and analyzed a community-based registry that included information both about who witnessed cardiac arrest and who administered BCPR.

## METHODS

### Study design, population, and settings

This prospective, population-based observational study was carried out with an analysis of the Utstein Registry of the Fire and Disaster Management Agency in Niigata City, between 2012 and 2016. Niigata City is located on the northwest coast of Japan, with 800,000 inhabitants. This study included adult patients 18 years of age or older with witnessed OHCAs of medical origin. Medical origin was defined as cases in which the cause of the cardiac arrest is presumed to be cardiac or other medical cause (eg, anaphylaxis, asthma, and gastrointestinal bleeding), and in which there is no obvious cause of the cardiac arrest based on the international Utstein Style.^12^ We excluded OHCAs with a non-medical origin unwitnessed OHCAs, Emergency medical service (EMS)-witnessed OHCAs, OHCAs witnessed in medical facilities, and long-term care facilities, OHCA cases in which it was unknown who administered BCPR, and OHCA patients who had undergone transfer between hospitals from our analysis. This study was approved by the Institutional Ethics Review Board of Niigata City General Hospital (17-060), and the requirement for patient informed consent was waived.

### EMS system in Niigata City

The EMSs in Niigata City are two-tiered only when they are indicated for the protocol of physician-staffed ambulance service, which is available 24 hours a day.^13^ There are 2 tertiary care hospitals, 25 ambulances, and 1 physician-staffed ambulance in Niigata City. A standard ambulance comprises of 3 crewmembers, including at least 1 emergency life-saving technician (ELST). ELSTs are permitted to use advanced airways, intravenous line, and epinephrine administration only under on-line medical control direction.^14^

### Data collection

The following data were collected on a community-based scale, based on the international Utstein-style^12^: patient characteristics (sex and age), time of the day of cardiac arrest, location of cardiac arrest, type of person who witnessed a cardiac arrest, BCPR and bystander defibrillation with an automated external defibrillator (AED), type of person performing BCPR, dispatcher cardiopulmonary resuscitation (CPR) instruction, time course of resuscitation, cardiac arrest characteristics (first documented rhythm by EMS personnel and etiology of cardiac arrest), return of spontaneous circulation (ROSC) before EMS, ROSC prior to hospital arrival, 1-month survival, and neurologic status at 1 month after the event. The time of the day was divided into two categories: daytime (9:00–16:59) and night time (17:00–8:59).^10^^,^^15^ Locations of cardiac arrest were classified into three categories: residential area, public area (public buildings, workplace, streets/highway), and others, based on the preceding studies.^10^^,^^16^^,^^17^ Types of people who witnessed a cardiac arrest and who performed BCPR for the victims were divided into the following two groups: family members and non-family members (friends, colleagues, passersby, and others). Person who performed BCPR was investigated using observations by EMS or their interview at the scene. Shockable first rhythm was defined as either shock on application of AED by a bystander before EMS arrival or a first rhythm of ventricular fibrillation/ventricular tachycardia recorded by EMS.^16^ Neurological outcome was determined by inpatient-attending physicians using the Glasgow-Pittsburgh cerebral performance category scores 1-month post-OHCA. A cerebral performance category score of 1 (good performance) or 2 (moderate disability) was defined a favorable neurological outcome, and a cerebral performance category score of 3 (severe disability), 4 (vegetative state), or 5 (death) was defined a poor neurological outcome.^18^

### Outcome measures

The primary outcome was receiving BCPR.

### Statistical analysis

We compared the patient and EMS characteristics and outcomes dividing the included patients into the two groups (witnessed by family or non-family). Univariate analyses were performed with the use of a chi-squared test for categorical variables, and a Mann–Whitney U test for continuous variables.

First, we aimed to assess the association between the person who witnessed the arrest and the provision of BCPR using the multivariable analysis. Potential confounding factors based on biological plausibility and previous studies were included in the multivariable analysis.^10^^,^^15^ These variables included age (18–39, 40–64, or ≥65 years); sex (male or female); time of the day (daytime or nighttime); arrest location (residential, public, or others); and dispatcher instruction (yes or no).

BCPR performed by the witnessed person was defined as BCPR performed by the same type of person who witnessed the cardiac arrest. Among those who received BCPR, we sought to investigate the difference between the two groups in terms of the probability of receiving BCPR by a witnessed person, with the use of logistic regression models, adjusting for the same variables as mentioned above.

In addition, we assessed the disparities in the resuscitation process and outcomes between those witnessed by family and those by a non-family member in case of BCPR performed by a witnessing person using univariable logistic analysis. We assessed 1-month survival rate and neurological favorable outcomes using the multivariable logistic analysis adjusting for age (18–39, 40–64, or ≥65 years); sex; EMS response time; cardiac origin (yes or no); and initial shockable rhythm (yes or no).^19^^,^^20^ The threshold for significance was *P* < 0.05. All statistical analyses were conducted using SPSS version 23.0 (IBM Corporation, Armonk, NY, USA).

## RESULTS

**Figure 1.**
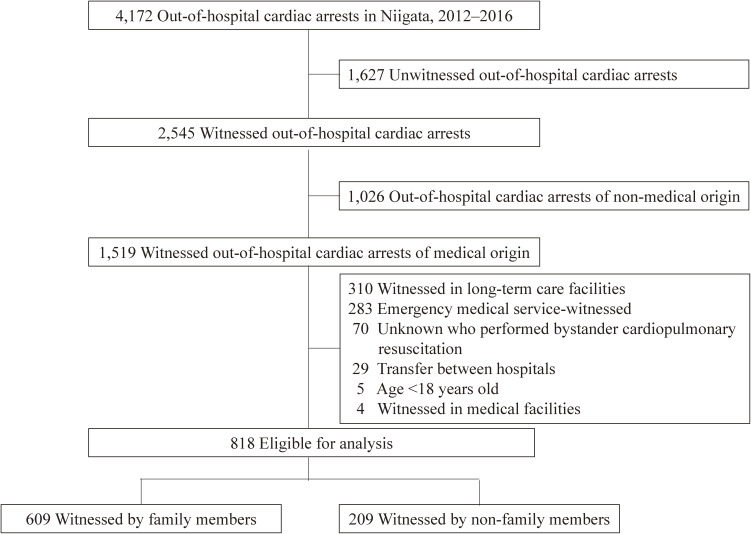
Patients with out-of-hospital cardiac arrests during the study period and patients included in the analysis

During the 5-year study period, resuscitation attempts had been performed in 4,172 cardiac arrests (Figure 1). Of 2,545 patients with witnessed OHCA, 1,519 had witnessed OHCA of a medical origin and, of these, 818 patients were eligible for our analyses.

### Patient characteristics

A total of 609 (74.4%) patients had been witnessed by family, whereas 209 (25.6%) patients had been witnessed by non-family members (Table 1). In the witnessed-by-family group, the patients were older; more patients experienced OHCA at night or in their residential area where a greater number of dispatcher instructions had been provided.

**Table 1.** Patient characteristics witnessed by a person

	Witnessed by family		Witnessed by non-family members		*P* values^a^
	*n* = 609	%	*n* = 209	%	
Age, years, median (IQR)	78 (67–85)		68 (55–83)		<0.001
Age category, years					<0.001
18–39	16	2.6	13	6.2	
40–64	106	17.4	77	36.8	
≥65	487	80.0	119	56.9	
Female sex	213	35.0	59	28.2	0.074
Time of day					<0.001
daytime (9:00–16:59)	185	30.4	120	57.4	
nighttime (17:00–8:59)	424	69.6	89	42.6	
Location					<0.001
Residential	563	92.4	47	22.5	
Public	25	4.1	144	68.9	
Others	21	3.4	18	8.6	
Dispatcher instruction	312	51.2	83	39.7	0.004
EMS response time,^b^ min	8 (6–10)		8 (6–9)		0.028
Cardiac origin	345	56.7	155	74.2	<0.001
Shockable rhythm	111	18.2	75	35.9	<0.001

IQR, interquartile range; EMS, emergency medical service.

^a^Comparison between the two groups was evaluated with the Mann–Whitney U test for continuous variables and the chi-squared test for categorical variables.

Missing data.

^b^*n* = 2 (0.2%).

### Association between BCPR and the witnessed person

Overall, OHCA patients witnessed by family were less likely to receive BCPR compared to those witnessed by non-family members (42.7% [260/609] versus 56.9% [119/209], *P* = 0.017) (Table 2). In addition, among the witnessed patients for whom BCPR was performed, the proportion of BCPR performed by a witnessing family member was less than that performed by a witnessing non-family member (93.1% [242/260] versus 97.5% [116/119], *P* = 0.011) (Table 3).

**Table 2.** Bystander cardiopulmonary resuscitation according to the witnessing person

	Total	Bystander CPR	%	*P* value^a^
Witnessed CPR				
Family	609	260	42.7	0.017
Non-family	209	119	56.9	

CPR, cardiopulmonary resuscitation.

^a^Adjusted for age, sex, time of day, location, and dispatcher instruction.

**Table 3.** Association between witnessing family and non-family members among the patients who received bystander cardiopulmonary resuscitation performed by the witnessing person

	Total	BCPR by witnessing person	%	BCPR by non-witnessing person	%	*P* value^a^
Patients who received BCPR						0.011
Family witness	260	242 (family BCPR)	93.1	18 (non-family BCPR)	6.9	
Non-family witness	119	116 (non-family BCPR)	97.5	3 (family BCPR)	2.5	

BCPR, bystander cardiopulmonary resuscitation.

^a^Adjusted for age, sex, time of day, location, and dispatcher instruction.

Regarding the disparities in the resuscitation process and outcomes between those witnessed by family and those by non-family members in the case of BCPR performed by a witnessing person, in the family group, the median time from the cardiac pulmonary arrest (CPA) to starting the BCPR was longer (3 minutes [IQR 1–5] vs 2 minutes [IQR 0–3], *P* < 0.001); lesser people used a bystander AED (0.8% [2/242] vs 14.7% [17/116], *P* < 0.001); and fewer patients were ROSC before EMS despite more dispatcher instruction (0.4% [1/242] vs 5.2% [6/116], *P* = 0.002) (Table 4). In addition, a greater proportion of the non-family witnessed group started BCPR before the EMS call (5.8% [14/242] vs 13.8% [16/116], *P* = 0.011). Furthermore, the non-family witnessed group had a greater proportion of those with 1-month survival with a favorable neurological outcome (9.9% [24/242] vs 30.2% [35/116], *P* = 0.012).

**Table 4.** Differences between bystander cardiopulmonary resuscitation by family and non-family members among patients who received bystander cardiopulmonary resuscitation performed by a witnessing person

	Bystander CPR by a witnessing person				
	Family		Non-family		*P* values^a^
	*n* = 242	%	*n* = 116	%	
Age, years, median (IQR)	77 (66–85)		69 (55–83)		0.002
Age category, years					<0.001
18–39	8	3.3	8	6.9	
40–64	45	18.6	43	37.1	
≥65	189	78.1	65	56.0	
Female sex	102	42.1	32	27.6	0.008
Started bystander CPR before EMS call^b^	14	5.8	16	13.8	0.011
Dispatcher instruction	215	88.8	73	62.9	<0.001
Time from CPA to start of bystander CPR,^c^ min	3 (1–5)		2 (0–3)		<0.001
Bystander AED	2	0.8	17	14.7	<0.001
EMS response time	9 (7–11)		9 (7–11)		0.590
Cardiac cause	146	60.3	86	74.1	0.010
Shockable rhythm	60	24.8	45	38.8	0.006
ROSC before EMS	1	0.4	6	5.2	0.002
ROSC prior to hospital arrival	60	24.8	39	33.6	0.081
1-month survival^d^	33	13.6	39	33.6	0.024
Neurologic favourable survival^d^	24	9.9	35	30.2	0.012

CPR, cardiopulmonary resuscitation; IQR, interquartile range; EMS, emergency medical service; CPA, cardiac pulmonary arrest; AED, automated external defibrillator; ROSC, return of spontaneous circulation.

^a^Comparison between the two groups was evaluated with the Mann–Whitney U test for continuous variables and the chi-squared test for categorical variables.

Missing data.

^b^*n* = 15 (4.2%), ^c^*n* = 61 (17.0%).

^d^Adjusted for age, sex, EMS response time, cardiac origin, and shockable rhythm.

## DISCUSSION

This community-based observational study showed that a witnessing family member was less likely to perform BCPR than a non-family member. Our data also demonstrated that the proportion of BCPR performed by a witnessing family member was significantly lower than that performed by a non-family member. This study is, to our knowledge, the first study to report these outcomes, and our findings should be helpful for increasing the number of BCPRs.

In this study, OHCAs witnessed by family members were less likely to receive BCPR than those witnessed by non-family members. The results of this study support the findings of a prior study.^11^ According to nationwide data between 2005 and 2009 in Japan, 37.8% of patients witnessed by family members received BCPR, while 43.7% of patients witnessed by friends or colleagues received BCPR and 59.3% of patients witnessed by other people received BCPR.^11^ The rate of the performance of BCPR by family members was relatively high in our study (42.9% of family members vs 56.7% of non-family members). The plausible explanation for this is the accumulation of citizens trained in CPR in the population and continuous efforts of the EMS system.^4^ In addition, the increase in bystander CPR might be due in part to the change in CPR guidelines that accept CPR using only chest compression.^21^ Although this previous study did not include the data of people who actually administered the BCPR, our study included information about both, those who witnessed and those who actually administered BCPR. The present study can provide an actual situation of cardiac arrests at the scene, which would enable us to perform detailed assessment of the barrier of performing BCPR.

We analyzed the data regarding those who actually performed BCPR and found that the proportion of BCPR performed was lower in those witnessed by family members than in those witnessed by non-family members. The underlying mechanism of our findings may be multifactorial. First, a witnessing family may suffer emotional stress, particularly when they witness the sudden collapse of their family member.^11^^,^^22^ Second, there may be psychological barriers to overcome to perform CPR on a known victim.^23^ Third, a bystander or rescuer might be present alone when a family member experiences a sudden cardiac arrest because most of events occur in residential areas and the average number of household members in Japan is decreasing, being at 2.44 people/house in 2018.^24^ Furthermore, the proportion of elderly people who have to care for their elderly spouse has been increasing with aging society of Japan.^24^ Therefore, bystanders for elderly patients might be also elderly in residential location. It would be hard for elderly person to provide BCPR.

We assessed not only the performance of BCPR by a witnessed person but also the disparities in the resuscitation process and their outcomes. In BCPR performed by a witnessing non-family member, it took a shorter time from CPA to the start of bystander CPR despite less dispatcher instruction. Even family members who performed BCPR started it later than non-family members or after dispatcher instruction. There may be obstacles for family members to perform BCPR. As a result, those who received BCPR from a non-family witness may be more likely to have a first documented shockable rhythm, achieve ROSC before EMS, and survive with a more favorable neurological status. Another important mechanism might be that reduced use of defibrillator or longer time to defibrillation in the family member is likely to be related to the significant difference in location of arrest; 92.5% of family-witnessed arrests were in residential setting and more commonly out of hours.

This study has several clinical, research, and public health implications. First, the observed difference in bystander-patient relationship should be a target of quality improvement efforts to increase the provision of BCPR for OHCA. Our findings demonstrated that family members were less likely to perform CPR. Considering the fact that a family encounters three times as many witnessed OHCAs of medical origin as non-family members in this study, further intervention for the majority are needed. In fact, CPR training for family members of individuals with cardiac diseases is effective and targeted training of high-risk cardiac groups has been advocated and actually guideline recommended the training for specific population.^2^^,^^25^^–^^28^ Public awareness about the importance of BCPR among family members needs to be broadened nationwide. In addition, dispatcher instruction for families, considering their emotional stress or psychological barriers, should be provided. Second, our results justify further efforts to identify underlying reasons for this observed bystander–patient relationship difference in performing BCPR and address them. Further research to overcome the barriers should be performed.

### Limitations

Our study has some limitations. First, no data on BCPR quality were collected. Second, our findings may not be fully generalizable to other healthcare settings, considering the differences in patient characteristics and medical care systems. Third, we did not measure the patients’ underlying diseases or comorbidities, or detailed basic demographics of BCPR providers, such as age, sex, relation or history of CPR training. Finally, although this observational study was adjusted for covariates as far as possible, we could not exclude possible residual confounding factors.

### Conclusion

In this community-based observational study, we found that a witnessing family member is less likely to perform BCPR than a witnessing non-family member. Further efforts on education and research to overcome the barriers for family members to perform BCPR are needed.
